# Supplementary material for: Systematic multivariate analysis of chromatin complex dependencies reveals Set1C/COMPASS as a melanoma-enriched epigenetic vulnerability
Source: PLoS Comput Biol. 2026 Aug 27;22(8):e1014018. doi: 10.1371/journal.pcbi.1014018 (PMC13537691; doi:10.1371/journal.pcbi.1014018)
Supplement: Supplementary file 2 [file pcbi.1014018.s009.docx]

***Response to Review of PCOMPBIOL-D-26-00353***

***“Systematic multivariate analysis of chromatin complex dependencies reveals Set1C/COMPASS as a melanoma-enriched epigenetic vulnerability”***

We thank the reviewers for their constructive comments and suggestions to improve the manuscript. We have addressed all the comments in the revised manuscript. Below, we provide a point-by-point response to the reviewers’ comments, outlining the revisions we have made to address them. We believe that with these revisions, the manuscript has been significantly improved.

**Additional Editor Comments:
Reviewers agreed that the study was rigorous and interesting. Multiple reviewers noted that including more rationale for decisions and altering some discussion points is warranted.**

As detailed below, we have addressed all the reviewers’ comments, provided clear justifications for the decisions made, and revised the Discussion as recommended by the reviewers.

**Reviewer #1:**

**In this study, Quesada Camacho and Fallahi-Sichani have systematically identified lineage-enriched epigenetic vulnerabilities across many cancer types by bioinformatic analysis of public datasets. In melanoma, they identified a novel dependence on the H3K4 methyltransferase complex Set1C/COMPASS. Using a panel of melanoma cell lines, the authors show that some but not all cell lines are dependent on CXXC1 with dependent cell lines showing reduced H3K4 trimethylation and cell cycle progression upon CXXC1 depletion by siRNA. They also show that CXXC1-dependent cell lines show alterations in E2F and MYC pathways upon CXXC1 depletion.**

**Overall, the study is well done and carefully controlled. The bioinformatic analysis is well done, and the experiments with CXXC1 knockdown are very thorough. In addition, the top-level finding that some but not all melanomas are dependent on the Set1C/COMPASS complex would be novel.**

We appreciate the reviewer's positive assessment of our study.

**However, there are several weaknesses that the authors should address to strengthen their findings:
- CXXC1 is a member of both the SETD1A and SETD1B COMPASS complexes. Thus, the data demonstrating that some cell lines are CXXC1-dependent does not distinguish between SETD1A and SETD1B. The authors should add experimental demonstration that the CXXC1-dependent cell lines are also sensitive to SETD1B knockdown.**

To address this point, we examined the relationship between CXXC1 and SETD1B dependency in melanoma cell lines (assessed by CRISPR knockout) using two complementary approaches. First, pairwise correlation analysis across 67 melanoma cell lines revealed a statistically significant correlation between CXXC1 and SETD1B dependency (Pearson’s *r* = 0.41, *P* = 5.2×10^-4^; revised Figure S1B). Second, comparison of SETD1B dependency scores between subgroups of melanoma cell lines stratified by CXXC1 dependency (CXXC1^Low^ vs CXXC1^High^) showed a significantly higher SETD1B dependency in the CXXC1^High^ group (*P* = 1.4 × 10^-3^; revised Figure 2G). In contrast, neither analysis identified a significant association between CXXC1 and SETD1A dependency (revised Figures S1A and 2F). These results support a correlated relationship between CXXC1 and SETD1B dependency, but not SETD1A, in melanoma cells. It’s noteworthy

**- Despite the careful characterization of the effects of CXXC1 knockdown, there does not appear to be any way to identify CXXC1-dependent cell lines. In other words, what are the biomarkers of CXXC1-dependence? Or is it impossible to know until you knock down CXXC1 and measure the response?**

This is an important question given the heterogeneity of CXXC1 dependency across melanoma cell lines. To address this, we performed several analyses to identify potential biomarkers associated with CXXC1 dependency. We first examined associations with genetic alterations in Set1C/COMPASS subunits (revised Figures S1C-D) and melanoma differentiation state (revised Figure 3), but neither showed a robust and significant relationship with CXXC1 dependency. We next performed a transcriptome-wide analysis to identify transcriptional programs associated with Set1C/COMPASS dependency. This analysis revealed significant enrichment of multiple pathways, most notably the expression of MYC and E2F target gene programs (revised Figure 6A). Importantly, these same pathways also exhibited increased genetic dependency in CXXC1-dependent melanoma cell lines (revised Figure 6B) and were downregulated following CXXC1 depletion in H3K4me3-responsive cell lines, but not in H3K4me3-nonresponsive cell lines (revised Figure 7). Thus, the strongest biomarkers identified in our current study are elevated expression of, and dependency on, MYC- and E2F-driven transcriptional programs. However, in response to the reviewer’s comment, we also explored whether more direct epigenetic biomarkers could be identified using publicly available datasets. We found no significant association between CXXC1 dependency and the expression of individual Set1C/COMPASS subunits (revised Figure S1E). In addition, we systematically analyzed global histone post-translational modifications quantified by mass spectrometry across CCLE cell lines and identified a significant correlation between H3K27ac1K36me0 abundance and CXXC1 dependency (revised Figure S1F). However, the robustness and mechanistic basis of this association remain to be established. Therefore, although our analyses identify transcriptional programs associated with CXXC1 dependency, identifying direct mechanistic predictors of this vulnerability will require further investigation. We have added this discussion to the revised manuscript.

**- The epigenetic complex dependence analysis done by cancer lineage (Fig. 1) misses dependencies that are unique to cancer sub-types (e.g., BRCA can be divided into luminal A/B, HER2-positive, and basal). Have the authors explored whether there are sub-type-dependent epigenetic dependencies for those tumor types where there are sufficient number of cell lines?**

We did not systematically extend our analysis of epigenetic complex dependencies to cancer subtypes for two main reasons. First, the number of available cell lines varies substantially across cancer subtypes, resulting in limited statistical power and less robust conclusions for many subtype-specific analyses. Second, our study was primarily motivated by identifying epigenetic dependencies associated with biological contexts that are rooted in epigenetic and transcriptome-wide differences, such as tissue lineage and cellular differentiation state. However, cancer subtype definitions are not standardized across malignancies and often reflect distinct biological features. For example, some breast cancer molecular subtypes are defined by receptor status or specific genetic alterations (e.g., HER2 amplification or hormone receptor positivity), whereas melanoma subtypes (beyond BRAF/RAS mutation status) are defined by differentiation state. Because differentiation-state annotations are not available in the CCLE database, they had to be inferred from transcriptomic data analysis in our study and require extensive additional analyses beyond what’s available in CCLE annotations. Accordingly, for melanoma (the primary focus of our work), we systematically evaluated epigenetic dependencies across well-defined differentiation states, melanocytic-like and undifferentiated/neural crest-like (revised Figure 3). The same analytical framework could be extended to other cancer types (in future studies) by first defining lineage-specific differentiation-state gene signatures, classifying cell lines based on transcriptomic profiles, and then evaluating epigenetic complex dependencies within these biologically defined states.

**- The variance explained by PC4 in Fig. 6B is only 2.7%. Can the authors comment on or justify why the effect of CXCC1 knockdown is so small relative to cell type effects?**

The relatively low variance explained by PC4 (2.7%) is expected given the design of the analysis. The PCA was performed jointly on four genetically distinct melanoma cell lines that were intentionally selected to span a broad range of differentiation states. Consequently, the first three principal components, which together explain ~80% of the total variance, primarily capture the substantial genetic and transcriptional differences and differentiation-state heterogeneity among four cell lines. In contrast, CXXC1 depletion represents a single perturbation superimposed on these markedly different transcriptional backgrounds and is therefore expected to account for a substantially smaller fraction of the overall variance. Importantly, the relatively modest variance explained by PC4 relative to PC1-PC3 does not imply a weak biological effect. Rather, PC4 selectively captures the transcriptional response to CXXC1 depletion in H3K4me3-responsive cell lines, which as demonstrated in revised Figures 7C-G, reflects coordinated changes in hundreds of genes that are enriched for a limited set of biological pathways, including MYC- and E2F-driven transcriptional programs. Given that the transcriptome comprises >20,000 protein-coding genes, coordinated change in approximately 2-3% of expressed genes is ot inconsistent with a principal component accounting for only 2.7% of the total transcriptomic variance.

**- The authors use two somewhat arbitrary thresholds in the analysis of VIP scores in Fig. 2C (0.6 and 1.0). Is there a threshold-free analysis that could be done to reduce the reliance on these thresholds?**

To avoid any confusion, we removed the secondary |VIP| threshold of 0.6 from the analysis (see revised Figure 3C). It is important to note, however, that the |VIP| threshold of 1 is not arbitrary. VIP scores are normalized such that the average squared VIP score across all predictors equals 1. Consequently, if all variables contributed equally to the predictive performance of the model, each would have a VIP score of 1. A VIP score greater than 1 therefore indicates that a variable contributes more than average to explaining the response, whereas a score below 1 indicates a below-average contribution. By removing the secondary |VIP| threshold of 0.6 and relying only on the statistically meaningful threshold of |VIP| ≥ 1, our analysis indicates that Set1C/COMPASS dependency is not associated with either melanoma differentiation state, as all Set1C/COMPASS subunits except ASH2L have |VIP| values below 1. This conclusion is further supported by univariate comparisons of dependency scores, which showed no statistically significant differences between melanocytic-like and undifferentiated/neural crest-like cell lines for any Set1C/COMPASS subunit other than ASH2L (revised Figure 3F).

**Minor Points
• Figure 6E has a typo in the title**

Thank you for noticing this typo. We have corrected it in the revised figure.

**Reviewer #2:**

**This paper leverages large public datasets to investigate epigenetic differences across cell lines representing many different types of cancer. This work is relevant because the ways in which epigenetic mechanisms regulate specific cancer types is not well studied. Analyses of the datasets identify a lineage-specific epigenetic vulnerability in melanoma that is independent of the melanoma differentiation state. Specifically, the authors find that the methyltransferase complex Set1C/COMPASS involving the CXXC1 subunit of SETD1B influences cell proliferation and transcriptional programs in CXXC1-dependent melanoma cell lines.

Overall, the paper provides relevant and interesting findings, the methods are rigorous and well justified, and the work is presented clearly. However, some aspects of the paper should be refined to improve the study.**

We appreciate the reviewer's positive assessment of our study and we have addressed the comments by revising the manuscript as described below.

**Specific comments:
- My main concern is related to justifying the results and analyses presented. This is reflected in a number of ways:
1. Although the initial analysis included 42 cancer types, the paper almost immediately focused on melanoma. Greater justification is needed here, as many threads could be pulled on given the rich results shown in Fig. 1A. Why did melanoma specifically become the focus?**

We agree with the reviewer that the transition from the pan-cancer analysis to the melanoma-focused analyses in the original manuscript could be made clearer. Indeed, the primary objective of this study was to identify novel epigenetic dependencies in melanoma, motivated by: (a) previous work from our group and others, (b) the well-characterized transcriptional heterogeneity of melanoma across differentiation states, and (c) prior evidence implicating diverse chromatin regulators in melanoma biology as described in detail in the Introduction. Accordingly, we adopted a systematic, multivariate pan-cancer approach to identify epigenetic dependencies that are selectively enriched in the melanoma lineage relative to other cancer types, as well as across distinct differentiation states within melanoma. Thus, the focus on melanoma was established from the outset. However, as part of our initial approach, the pan-cancer analysis identified other lineage-specific epigenetic vulnerabilities and as the reviewer noted, Figure 1A contains many additional findings that could be explored further across diverse cancer lineages. Investigating all of these potentially interesting findings was beyond the scope of the present study. Therefore, we focused on the melanoma-specific vulnerability through additional computational analyses and experimental validation to uncover its associated mechanisms. We nevertheless hope that the new findings highlighted by our pan-cancer analysis will provide a useful resource for future studies. To clarify this rationale, we have revised multiple parts of the manuscript, including the Introduction and the opening paragraph of the Results, to more clearly explain the motivation for the pan-cancer analysis as well as transition to the melanoma-focused analyses.

**2. Similar to melanoma, in Fig. 1A, MBN and AML also have a strong dependency on Set1C/COMPASS. Should this be further investigated? Some discussion is needed here.**

Our analysis identified statistically significant enrichment of Set1C/COMPASS dependency in a couple of other cancer lineages in addition to melanoma (MEL), including B-cell acute lymphoblastic leukemia (B-ALL) and head and neck squamous cell carcinoma (HNSC). These have been discussed in multiple points in the manuscript, including the last section of Results and the revised Figure S5. Furthermore, there is experimental evidence regarding the role of Set1C/COMPASS as an epigenetic vulnerability in AML that we have discussed in the Results and Discussion. The Figure 1A dependencies noted by the reviewer for mature B-cell neoplasms (MBN) and acute myeloid leukemia (AML) appear to be related to other H3K4 methyltransferase complexes (including MLL1/MLL2, MLL3/MLL4, and Menin-MLL) that were enriched in hematopoietic malignancies.

**3. Is it meaningful that SETD1B and CXXC1 are only considered important contributors to the epigenetic gene-dependency when the VIP threshold is relaxed? More justification is needed.**

To address the reviewer’s concern and to avoid any confusion, we removed the secondary |VIP| threshold of 0.6 from the analysis (see revised Figure 3C). VIP scores are normalized such that the average squared VIP score across all predictors equals 1. Consequently, if all variables contributed equally to the predictive performance of the model, each would have a VIP score of 1. A VIP score greater than 1 therefore indicates that a variable contributes more than average to explaining the response, whereas a score below 1 indicates a below-average contribution. By removing the secondary |VIP| threshold of 0.6 and relying only on the statistically meaningful threshold of |VIP| ≥ 1, our analysis indicates that Set1C/COMPASS dependency is not associated with either melanoma differentiation state, as all Set1C/COMPASS subunits except ASH2L have |VIP| values below 1. This conclusion is further supported by univariate comparisons of dependency scores, which showed no statistically significant differences between melanocytic-like and undifferentiated/neural crest-like cell lines for any Set1C/COMPASS subunit other than ASH2L (revised Figure 3F).

**4. When silencing CXXC1, the CXXC1-dependent cell line LOXIMVI does not show differences in Ki67 to the same extent as the other CXXC1-dependent lines, but does show differences in the percentage of Ki67-high cells compared to control (Fig. 3A and H). This cell line is also an outlier in Fig. 3J. The authors should explain this.**

To address this comment, we performed additional statistical analyses and revised the description of Results for this section to more clearly evaluate the effects of CXXC1 siRNA treatment and distinguish the different response patterns observed across the five melanoma cell lines, including the more moderate effect of CXXC1 depletion on LOXIMVI relative to other sensitive cell lines. We also revised the way melanoma cell lines were classified and instead of assigning cell lines to CXXC1-dependent (sensitive) and CXXC1-independent (insensitive) cell lines, we assigned them to H3K4me3-responsive and H3K4me3-nonresponsive groups based on careful statistical analysis as described below.

Across all cell lines, CXXC1 siRNA resulted in significant depletion of CXXC1 protein relative to non-targeting control (FDR < 0.05) (revised Figures 4C and S3A-B). Although the magnitude of knockdown varied across cell lines, every cell line exhibited more than a 30% reduction in the fraction of CXXC1^High^ cells following CXXC1 siRNA treatment relative to non-targeting control. Taking into account the differences in baseline CXXC1 expression, the estimated knockdown efficiency ranged from approximately 75% to 95%, indicating robust CXXC1 depletion in all five cell lines (revised Table S2A). However, only three cell lines, including MALME3M, UACC62, and LOXIMVI, exhibited a significant (FDR < 0.05) and greater than 30% reduction effect in the fraction of H3K4me3^High^ cells (revised Figures 4D and S3C-D and revised Table S2B). By comparison, SKMEL28 showed only a modest reduction (~4%), whereas WM115 had a low baseline fraction of H3K4me3^High^ cells that did not change significantly (FDR = 0.4) following CXXC1 depletion. Based on these statistically different H3K4me3 responses following CXXC1 knockdown, we classified MALME3M, UACC62, and LOXIMVI as H3K4me3-responsive cell lines, and SKMEL28 and WM115 as H3K4me3-nonresponsive cell lines (Table S2B).

Next, using normalized cell count as a bulk readout of cellular growth 96 h following siRNA treatment, we found that two of the H3K4me3-responsive cell lines (MALME3M, UACC62) exhibited the strongest reductions in normalized cell count (~21% and ~53% reduction with effect size of ~24 and ~19, respectively) that were statistically significant (FDR < 0.05), whereas the H3K4me3-nonresponsive cell lines SKMEL28 and WM115 showed the weakest effect sizes (-1.8 and ~0.1, respectively) that were not significant (FDR = ~0.9 and ~0.6, respectively; revised Figure 4E and revised Table S2C). For LOXIMVI (the third H3K4me3-responsive cell line), we observed only a modest reduction in normalized cell count (~16%) with an effect size of 2.25 that did not meet the FDR cutoff for statistical significance (FDR = 0.09; Table S2C). Therefore, the modest response of LOXIMVI is not at the level of the other two H3K4me3-responsive cell lines (MALME3M, UACC62) but its effect size is greater than H3K4me3-nonresponsive cell lines.

These results suggest a correspondence between the H3K4me3 response to CXXC1 depletion and CXXC1 dependency, as measured by the effect of CXXC1 knockdown on bulk cellular growth. Consistent with this, interaction tests confirmed that the effects of CXXC1 knockdown on both H3K4me3 and normalized cell count differed significantly (*P* ≈ 0.03 and *P* ≈ 0.002, respectively) between H3K4me3-responsive (MALME3M, UACC62, and LOXIMVI) and H3K4me3-nonresponsive (SKMEL28 and WM115) cell lines (revised Table S2D), supporting an association between H3K4me3 response and CXXC1 dependency. Accordingly, siRNA-induced changes in H3K4me3 were correlated with changes in normalized cell count across all five cell lines (revised Figure 4F).

We next asked whether the concurrent depletion of H3K4me3 and reduction in bulk cellular growth were accompanied by changes in the proliferative state of melanoma cells, as measured by Ki-67 positivity at the single-cell level. MALME3M and UACC62, the two cell lines exhibiting the greatest reductions in both H3K4me3 and normalized cell count following CXXC1 depletion, also showed the largest decreases (approximately 50%) in the fraction of Ki-67^High^ cells (revised Figures 4G and S3E-F). In contrast, LOXIMVI, as well as the two H3K4me3-nonresponsive cell lines SKMEL28 and WM115, exhibited only modest reductions (<20%) in the fraction of Ki-67^High^ cells (revised Figures 4G and S3E-F). Consistent with these data, MALME3M also showed a marked reduction in phosphorylation of Rb at Ser807/811 and Thr373, additional indicators of cell-cycle progression, whereas LOXIMVI exhibited a moderate effect and SKMEL28 showed no significant change in Rb phosphorylation following CXXC1 depletion (revised Figures S3G-H).

**5. Related, the LOXIMVI cell line is not considered in the analyses presented in Fig. 4. What is the justification for this?**

The purpose of the single-cell analysis presented in Figure 4 of the original manuscript (revised Figure 5) is to determine whether the “robust” inhibition of proliferation observed following CXXC1 depletion occurs within the same cells that undergo H3K4me3 depletion. Accordingly, this analysis focused on the cell lines exhibiting a strongest and robust reduction in both normalized cell count and Ki-67, allowing us to determine whether the Ki-67^Low^ subpopulation corresponds to the H3K4me3^Low^ subpopulation through single-cell covariance analysis. Based on this criterion (and as discussed above), MALME3M and UACC62 (but not LOXIMVI) were the most appropriate cell lines, as they exhibited the largest decreases in both normalized cell count and Ki-67 following CXXC1 depletion (revised Figures 4E and 4G). We have added a clarifying sentence to the Results section to explain this.

**6. Only a portion of the transcriptional programs associated with Set1C/COMPASS dependency are highlighted in Fig. 5. What is the reasoning for this? Of particular interest are the programs related to EMT that are negatively associated (see comment below).**

**- I find it quite interesting that the EMT genes are negatively associated with Set1C/COMPASS dependency (Fig. 5; Fig. 6F,G). Related, there is also a negative association with TGFbeta and TNF-A, which are related to EMT. The authors should discuss this finding and is relevance in melanoma.**

We focused primarily on transcriptional programs that were positively associated with Set1C/COMPASS dependency at both the expression (revised Figure 6A) and functional dependency (revised Figure 6B) levels, as these programs are more likely to reflect direct consequences of Set1C/COMPASS activity. This rationale is based on the established role of Set1C/COMPASS in maintaining H3K4me3, a histone modification enriched at promoters of actively transcribed genes that promotes open chromatin and transcriptional initiation. Therefore, transcriptional programs that are negatively associated with Set1C/COMPASS dependency may represent indirect consequences rather than direct outputs of Set1C/COMPASS activity. As the reviewer noted, one such negatively associated program is the Hallmark Epithelial-Mesenchymal Transition (EMT) gene set, as well as several other gene sets (including KRAS signaling and IL-2/STAT5 signaling) that are now highlighted in the revised Figure 6 (green stars). Both the expression and dependency of these gene sets were negatively associated with Set1C/COMPASS dependency across melanoma cell lines. Regarding EMT genes, it’s noteworthy, however, that melanoma is not a canonical epithelial or mesenchymal cancer. Because it originates from the neural crest lineage, cellular plasticity in melanoma is typically described along a melanocytic-to-neural crest-like/undifferentiated (mesenchymal-like) trajectory rather than by classical EMT markers. Importantly, as shown in revised Figure 3, the association between Set1C/COMPASS dependency and the neural crest-like/undifferentiated state was relatively weak (VIP < 1), indicating that differentiation state itself is not a robust predictor of Set1C/COMPASS dependency. Regarding the TGF-β and TNFα signaling pathways highlighted by the reviewer, these showed negative association only at the gene expression level (revised Figure 6A), but not at the gene dependency level (revised Figure 6B), suggesting a weaker mechanistic relationship with Set1C/COMPASS activity. In the revised manuscript, we now discuss these negatively associated pathways while also explaining our rationale for emphasizing positively associated transcriptional programs, which are supported by both expression and functional dependency analyses and are therefore more likely to reflect direct biological outputs of Set1C/COMPASS activity.

**- As written, the single-cell analysis is disconnected from the other analyses. Can the authors better integrate these findings in the narrative? Is the greater cell-to-cell heterogeneity for different markers meaningful? How can the insights from single cells be leveraged?**

The population-level analyses in revised Figure 4 show that CXXC1 depletion reduces H3K4me3 and impairs proliferation in the most sensitive cell lines, but they cannot determine whether these responses occur within the same cells or reflect independent effects in distinct subpopulations in these cell lines. The single-cell analyses described in the revised Figure 5 address this question by simultaneously quantifying CXXC1 and H3K4me3, or H3K4me3 together with proliferation markers, within individual cells. The covariate single-cell analyses show that cells exhibiting the greatest reduction in the proliferation markers Ki-67 or phosphorylated Rb are highly enriched within the H3K4me3^Low^ subpopulation generated following CXXC1 depletion. Moreover, single-cell analysis of phosphorylated Rb enables cell-cycle phase assignment, showing that loss of H3K4me3 is associated with G1 arrest (revised Figure 5D), an insight that could not be obtained from the population-level analyses alone. We have revised the opening and interpretation of the Results section describing Figure 5 to better clarify these points.

**- Minor comment: Perhaps I missed it, but what do the colors in the labels along the vertical and horizontal axes in Fig. 1A correspond to? These should be explained.**

We had described the color annotations in the Results section when discussing Figure 1A, but have now updated the Figure 1A legend to include the same information. Column labels are colored to highlight functionally related clusters of epigenetic complexes: red, H3K4 methyltransferase complexes; blue, SWI/SNF-family chromatin remodelers; purple, ISWI-family chromatin remodelers; orange, SAGA-type histone acetyltransferase complexes. Row labels colored in brown represent several hematopoietic malignancies (acute leukemia/B-cell neoplasms) that were clustered together based on their shared epigenetic dependencies. The column label for the melanoma lineage is shown in green.

**Reviewer #3:**

**This manuscript presents a well-executed, integrative computational and experimental study identifying Set1C/COMPASS as a melanoma-enriched epigenetic vulnerability. The authors systematically analyze DepMap CRISPR dependency data across 42 cancer types, combined with curated chromatin complex annotations, to uncover lineage-specific dependencies at the complex level. They validate their findings through targeted siRNA experiments, multiplexed immunofluorescence imaging (4i), single-cell analysis, and RNA-seq. The work is timely, methodologically sound, and addresses an important gap in understanding context-dependent epigenetic dependencies in cancer. However, several issues regarding methodological transparency, statistical rigor, and presentation clarity should be addressed before publication.**

We appreciate the reviewer's positive assessment of our study, and we have addressed the comments by revising the manuscript as described below.

**1. The finding that Set1C/COMPASS supports MYC-driven transcriptional programs in cancer is not entirely new (see Izumi et al., Leukemia 2025, cited by authors). While the authors acknowledge this, the novelty would be strengthened by more explicitly delineating what is uniquely melanoma-specific about this dependency versus a more general phenomenon across SETD1B-dependent cancers.**

This is an interesting point raised by the reviewer. After identifying and experimentally validating the association between Set1C/COMPASS dependency and MYC- and E2F-driven transcriptional programs in melanoma, we searched the literature for similar observations in other cancer types. As the reviewer noted, we found a recent study by Izumi et al. (2025) in acute myeloid leukemia (AML), which reported that SETD1B-dependent H3K4 methylation is required to sustain MYC-driven cellular proliferation. Based on these complementary findings, we suggested in the Discussion that Set1C/COMPASS dependency in both melanoma and AML may reflect a common requirement to maintain H3K4 trimethylation in support of MYC- and E2F-driven transcriptional programs.

However, the reviewer’s comment prompted us to examine this relationship more systematically across cancer lineages. Although our large-scale pan-cancer analysis (Figure 1A) identified significant enrichment of Set1C/COMPASS dependency in only a few cancer types, including melanoma (MEL), B-cell acute lymphoblastic leukemia (B-ALL), and head and neck squamous cell carcinoma (HNSC), many other lineages contained subsets of cell lines with elevated Set1C/COMPASS dependency. We therefore asked whether the association between Set1C/COMPASS dependency and MYC- and E2F-driven transcriptional programs is melanoma-specific or represents a more general feature of Set1C/COMPASS dependency across cancer lineages. To address this question, we extended the analysis presented in Figure 6 to all cancer lineages represented by at least five cell lines. For each lineage, we performed lineage-specific pre-ranked GSEA, ranking genes according to the correlation between their dependency scores and the Set1C/COMPASS dependency score across cell lines within that lineage. Remarkably, this association extended beyond melanoma, with most cancer lineages, including B-ALL, HNSC, and AML, exhibiting significant enrichment of MYC and E2F target gene dependencies among genes positively correlated with Set1C/COMPASS dependency (revised Figure S5). This association was particularly strong in HNSC, another cancer lineage showing significant enrichment of Set1C/COMPASS dependency in our pan-cancer analysis. Together, these data suggest that although the prevalence of Set1C/COMPASS dependency varies across cancer lineages and is statistically enriched in only a subset of cancers, its functional association with MYC- and E2F-driven transcriptional programs may be broadly conserved. However, the molecular determinants of Set1C/COMPASS dependency, and the basis for its differential enrichment across cancer lineages, remain to be elucidated. We have incorporated the additional analysis and the discussion in the revised manuscript.

**Additionally, the authors mention that "mutations in Set1C/COMPASS subunits are infrequent... and do not correlate with dependency scores" (Page 13-14, lines 8-10) — but do not explore whether expression levels of complex subunits or known regulators (e.g., Menin, WDR5-interacting proteins) correlate with dependency. This represents a missed opportunity to identify predictive biomarkers. Add an analysis correlating Set1C/COMPASS dependency with expression of complex subunits, known interactors, or chromatin states (e.g., baseline H3K4me3 levels) across melanoma cell lines. If no strong correlates exist, state this explicitly as an important unresolved question.**

As the reviewer suggested, we performed additional analyses correlating CXXC1 dependency in melanoma cell lines with the expression of all nine Set1C/COMPASS subunits (revised Figure S1E). We found no significant differences in the expression of any subunit. We also examined whether CXXC1 dependency correlates with baseline chromatin state using histone post-translational modification data available for CCLE cell lines. Unfortunately, H3K4me3 itself was not included in this dataset, but we did not observe an obvious relationship between baseline H3K4me3 levels and CXXC1 dependency across the five melanoma cell lines profiled experimentally in our study (revised Figure 4). The CCLE dataset includes other H3K4 methylation-related marks, including H3K4me0, H3K4me1, and H3K4me2, but none showed a significant association with CXXC1 dependency (revised Figure S1F). However, a systematic analysis of other histone post-translational modifications quantified by mass spectrometry across CCLE cell lines identified a significant correlation between H3K27ac1K36me0 abundance and CXXC1 dependency (revised Figure S1F). The robustness and mechanistic basis of this association, however, remain to be determined. Overall, we did not identify a strong or readily interpretable molecular correlate of CXXC1 dependency, and therefore the determinants of this vulnerability remain an important question for future investigation. We explicitly state this point in the Discussion of the revised manuscript.

**2. The authors classify cell lines as CXXC1-dependent based on normalized cell counts following siRNA treatment (Figure 3E). However, the threshold for "dependency" is not statistically defined. For LOXIMVI, the change appears modest (approximately 15-20% reduction), yet it is grouped with MALME3M and UACC62. What is the statistical justification? A formal interaction test (siRNA condition × cell line) or a predefined effect size threshold would improve rigor. Perform a two-way ANOVA with interaction term to formally test whether the effect of CXXC1 knockdown on cell count differs across cell lines. Report effect sizes (e.g., Cohen's d) and define dependency as a statistically significant reduction with effect size above a clinically/biologically meaningful threshold (e.g., >20% reduction, or FDR < 0.05 with |log2FC| > 0.3).**

As suggested by the reviewer, we performed additional statistical analyses (including interaction tests) and revised the presentation of Results section associated with the revised Figure 4 to more clearly evaluate the effects of CXXC1 siRNA treatment and distinguish the different response patterns observed across the five melanoma cell lines, including the more moderate effect of CXXC1 depletion on LOXIMVI relative to other sensitive cell lines (MALME3M and UACC62). We also have revised the way melanoma cell lines were classified and instead of assigning cell lines to CXXC1-dependent and CXXC1-independent cell lines, we assigned them to H3K4me3-responsive and H3K4me3-nonresponsive groups based on careful statistical analysis described below.

Across all cell lines, CXXC1 siRNA resulted in significant depletion of CXXC1 protein relative to non-targeting control (FDR < 0.05) (revised Figure 4C). Although the magnitude of knockdown varied across cell lines, every cell line exhibited more than a 30% reduction in the fraction of CXXC1^High^ cells following CXXC1 siRNA treatment relative to non-targeting control. Taking into account the differences in baseline CXXC1 expression, the estimated knockdown efficiency ranged from approximately 75% to 95%, indicating robust CXXC1 depletion in all five cell lines (see Table S2A, also shown below).

**Table S2A.** Statistical analysis of changes in the percentage of CXXC1^High^ cells following CXXC1 siRNA (si-CXXC1) treatment relative to non-targeting control (si-NTC) across five cell lines (n = 3 wells/biological replicates). Adjusted P values (FDR) were derived from linear mixed-effects models with treatment as a fixed effect and well/replicate as a random effect, using one-sided tests adjusted for multiple comparisons across cell lines using the Benjamini-Hochberg method.

| **Measurement** | **Cell Line** | **% Reduction** | **Effect size (Cohen's d)** | **FDR** | **Outcome** |
| --- | --- | --- | --- | --- | --- |
| % CXXC1^High^ | MALME3M | 77.0% | 20.31 | 1.55e-3 | Significant CXXC1 depletion |
| % CXXC1^High^ | UACC62 | 94.4% | 50.20 | 2.54e-6 | Significant CXXC1 depletion |
| % CXXC1^High^ | LOXIMVI | 75.0% | 15.24 | 1.55e-3 | Significant CXXC1 depletion |
| % CXXC1^High^ | SKMEL28 | 85.8% | 4.89 | 1.24e-2 | Significant CXXC1 depletion |
| % CXXC1^High^ | WM115 | 86.2% | 5.99 | 5.04e-3 | Significant CXXC1 depletion |

However, only three cell lines, including MALME3M, UACC62, and LOXIMVI, exhibited a significant (FDR < 0.05) and greater than 30% reduction effect in the fraction of H3K4me3^High^ cells (revised Figure 4D and Table S2B, shown below). By comparison, SKMEL28 showed only a modest reduction (~4%), whereas WM115 had a low baseline fraction of H3K4me3^High^ cells that did not change significantly (FDR = 0.4) following CXXC1 depletion. Based on these statistically different H3K4me3 responses following CXXC1 knockdown, we classified MALME3M, UACC62, and LOXIMVI as H3K4me3-responsive cell lines, and SKMEL28 and WM115 as H3K4me3-nonresponsive cell lines (Table S2B, also shown below).

**Table S2B.** Statistical analysis of changes in the percentage of H3K4me3^High^ cells following CXXC1 siRNA (si-CXXC1) treatment relative to non-targeting control (si-NTC) across five cell lines (n = 3 wells/biological replicates). Adjusted P values (FDR) were derived from linear mixed-effects models with treatment as a fixed effect and well/replicate as a random effect, using one-sided tests adjusted for multiple comparisons across cell lines using the Benjamini-Hochberg method.

| **Measurement** | **Cell Line** | **% Reduction** | **Effect size (Cohen's d)** | **FDR** | **Outcome** |
| --- | --- | --- | --- | --- | --- |
| % H3K4me3^High^ | MALME3M | 73.2% | 3.50 | 1.63e-2 | H3K4me3-responsive |
| % H3K4me3^High^ | UACC62 | 82.9% | 6.17 | 1.63e-2 | H3K4me3-responsive |
| % H3K4me3^High^ | LOXIMVI | 74.1% | 2.20 | 4.75e-2 | H3K4me3-responsive |
| % H3K4me3^High^ | SKMEL28 | 3.7% | 2.28 | 4.75e-2 | H3K4me3-nonresponsive |
| % H3K4me3^High^ | WM115 | 1.0% | 0.13 | 4.42e-1 | H3K4me3-nonresponsive |

Next, using normalized cell count as a bulk readout of cellular growth 96 h following siRNA treatment, we found that two of the H3K4me3-responsive cell lines (MALME3M, UACC62) exhibited the strongest reductions in normalized cell count (~21% and ~53% reduction with effect size of ~24 and ~19, respectively) that were statistically significant (FDR < 0.05), whereas the H3K4me3-nonresponsive cell lines SKMEL28 and WM115 showed the weakest effect sizes (-1.8 and ~0.1, respectively) that were not significant (FDR = ~0.9 and ~0.6, respectively; revised Figure 4E and Table S2C, shown below). For LOXIMVI (the third H3K4me3-responsive cell line), we observed only a modest reduction in normalized cell count (~16%) with an effect size of 2.25 that did not meet the FDR cutoff for statistical significance (FDR = 0.09; Table S2C, shown below). Therefore, the modest response of LOXIMVI was not at the level of the other two H3K4me3-responsive cell lines (MALME3M, UACC62) but its effect size was greater than H3K4me3-nonresponsive cell lines.

**Table S2C.** Statistical analysis of changes in the normalized cell count following CXXC1 siRNA (si-CXXC1) treatment relative to non-targeting control (si-NTC) across five cell lines (n = 3 wells/biological replicates). Adjusted P values (FDR) were derived from linear mixed-effects models with treatment as a fixed effect and well/replicate as a random effect, using one-sided tests adjusted for multiple comparisons across cell lines using the Benjamini-Hochberg method.

| **Measurement** | **Cell Line** | **% Reduction** | **Effect size (Cohen's d)** | **FDR** | **Outcome** |
| --- | --- | --- | --- | --- | --- |
| Normalized cell count | MALME3M | 21.1% | 24.19 | 2.23e-5 | Significant reduction of cell count |
| Normalized cell count | UACC62 | 52.8% | 18.77 | 7.60e-5 | Significant reduction of cell count |
| Normalized cell count | LOXIMVI | 16.1% | 2.25 | 9.01e-2 | Modest reduction of cell count |
| Normalized cell count | SKMEL28 | -8.9% (increase by 8.9%) | -1.83 (increase by 1.83) | 9.39e-1 | No reduction in cell count |
| Normalized cell count | WM115 | 1.0% | 0.12 | 5.56e-1 | No reduction in cell count |

These results suggest a correspondence between the H3K4me3 response to CXXC1 depletion and CXXC1 dependency, as measured by the effect of CXXC1 knockdown on bulk cellular growth. Consistent with this, as suggested by the reviewer, interaction tests confirmed that the effects of CXXC1 knockdown on both H3K4me3 and normalized cell count differed significantly (*P* ≈ 0.03 and *P* ≈ 0.002, respectively) between H3K4me3-responsive (MALME3M, UACC62, and LOXIMVI) and H3K4me3-nonresponsive (SKMEL28 and WM115) cell lines (Table S2D, shown below), supporting an association between H3K4me3 response and CXXC1 dependency. As summarized in the following Table, the interaction between siRNA condition and cell line was significant for both H3K4me3 and normalized cell count, indicating that the effect of CXXC1 knockdown varied among individual cell lines. When cell lines were grouped according to their H3K4me3 responsiveness, the interaction between siRNA condition and cell line group remained significant for both H3K4me3 and normalized cell count. However, the stronger interaction observed in the cell line analysis for normalized cell count is consistent with variability within the H3K4me3-responsive group, as LOXIMVI showed a more modest reduction in cell growth than MALME3M and UACC62.

**Table S2D.** Interaction analyses performed by extending the linear mixed-effects models to include cell line or cell-line group (H3K4me3-responsive versus H3K4me3-nonresponsive as determined in B) and their interaction with treatment. Satterthwaite F-tests were used to determine whether the effect of CXXC1 knockdown differed significantly across cell lines or cell-line groups.

| **Measurement** | **Interaction** | **F** | **df1** | **df2** | **p-value** |
| --- | --- | --- | --- | --- | --- |
| % H3K4me3^High^ | siRNA condition × cell line | 5.35 | 4 | 20 | 4.27e-3 |
| % H3K4me3^High^ | siRNA condition × cell line group | 5.26 | 1 | 26 | 3.01e-2 |
| Normalized cell count | siRNA condition × cell line | 34.85 | 4 | 20 | 9.42e-9 |
| Normalized cell count | siRNA condition × cell line group | 12.02 | 1 | 26 | 1.85e-3 |

All of the additional statistical analyses described above are included in the Results of the revised manuscript.

**3. The authors use Gaussian mixture models (GMMs) to binarize single-cell expression into "High" vs. "Low" states (Page 29, lines 2-8). This is appropriate, but the thresholds are derived by pooling data across conditions (si-NTC and si-CXXC1) within each cell line. This approach assumes that the underlying distribution components are stable across conditions, which may not hold if knockdown shifts the entire population. If the knockdown causes a uniform leftward shift without changing variance, the GMM threshold based on pooled data may misclassify cells. Either (a) fit separate GMMs for each condition and report classification consistency, or (b) use a fixed threshold based on the control condition only (e.g., 95th percentile of control, or mean + 2SD) and justify this choice. At minimum, perform a sensitivity analysis showing that main conclusions (Figures 3F-J, 4A-D) are robust to the thresholding method.**

We thank the reviewer for this comment. However, we would like to clarify that the Gaussian mixture model (GMM) thresholds were derived by pooling data across “all” cell lines and treatment conditions simultaneously for each measured protein marker, rather than fitting separate models to individual cell lines or experimental conditions. This global pooling approach uses the full range of variation present across the entire experiment to identify a single, stable classification threshold for each marker. The resulting threshold is then applied uniformly across all cell lines and treatment conditions to classify individual cells as High or Low, with the classification itself being blind to cell line identity and treatment condition. We believe this approach provides an unbiased classification strategy that does not rely on assumptions about the stability of distribution components within individual treatment groups. To better illustrate this approach, we now include the GMM fits for CXXC1, H3K4me3, and Ki-67, generated from the pooled data across all cell lines and treatment conditions, in the revised Figures S3B, S3D and S3F. We have also revised the Methods section to describe this classification procedure more clearly.

**4. The authors identify 26 epigenetic regulator genes with melanoma-enriched dependency using limma (FDR ≤ 0.05, mean difference > 0.1). This is appropriate. However, in Figure 1A, the complex-level analysis uses FDR ≤ 0.10 (Page 33, line 39) without justification for the more lenient threshold. The authors should either (a) apply the same FDR ≤ 0.05 threshold consistently, or (b) clearly justify the more lenient threshold for complex-level analysis (e.g., reduced statistical power due to averaging across subunits, or exploratory nature of complex-level discovery). Apply FDR ≤ 0.05 to complex-level analysis as a primary threshold. If key complexes (e.g., Set1C/COMPASS) no longer meet this threshold, present the FDR ≤ 0.10 results as "suggestive" or "nominal" and temper language accordingly in the Results and Discussion. Alternatively, provide power calculations demonstrating why FDR ≤ 0.10 is justified.**

The nominal FDR ≤ 0.10 threshold applied in the complex-level analysis was intentionally more permissive than the stringent FDR ≤ 0.05 threshold used in the gene-level analysis, and as described below, we now explicitly justify this distinction in the revised manuscript. Complex-level dependency scores are calculated by averaging the dependency scores of all annotated subunits within a complex, including subunits that may not individually contribute to a lineage-enriched dependency. This averaging can dilute true effect sizes and reduce statistical power, justifying the use of a more permissive threshold at this stage as an exploratory screen for candidate epigenetic complexes. Even with an FDR threshold of 0.10, only ~7% of lineage-specific complex dependencies were identified as significant. Applying a more stringent cutoff further reduced this number and failed to recover not only Set1C/COMPASS dependency, but also many other biologically well-established epigenetic dependencies. As noted, we subsequently evaluated the candidate complexes through an independent gene-level analysis using the more stringent FDR ≤ 0.05 threshold, followed by enrichment analysis. This second stage was designed to ensure that only robust gene-level associations were carried forward. Importantly, Set1C/COMPASS complex dependency was identified by both analyses, providing support for its enrichment in melanoma.

**5. A key claim is that CXXC1 dependency reflects Set1C/COMPASS activity (Page 15, lines 15-16: "To test whether dependency on CXXC1 in melanoma cells arises from its role in supporting Set1C/COMPASS activity"). However, the authors only show correlative evidence that CXXC1 depletion reduces H3K4me3 and proliferation. They do not test whether the growth defect is rescued by (a) overexpression of wild-type CXXC1, or (b) co-expression of other complex subunits (e.g., SETD1B) or (c) a catalytically inactive mutant (to distinguish scaffolding vs. enzymatic functions). Without rescue, off-target effects of siRNA cannot be definitively excluded. This is not necessarily required for publication in a computational journal, but the authors should acknowledge this limitation explicitly in the Discussion and tone down causal language (e.g., change "shows that CXXC1-dependent melanoma cells require Set1C/COMPASS activity" to "suggests that..." or "is consistent with..."). Alternatively, include at least one orthogonal validation (e.g., CRISPR knockout with complementation, or a second independent siRNA pool).**

We agree with the reviewer and have acknowledged this limitation explicitly in the Discussion, and we have toned down causal language according to the reviewer’s suggestion throughout the manuscript.

**6. The GSEA linking Set1C/COMPASS dependency to MYC/E2F targets (Figure 5) is compelling but correlative. The RNA-seq following CXXC1 knockdown (Figure 6) shows downregulation of these programs, but does not distinguish direct from indirect effects. ChIP-seq for H3K4me3 or CXXC1 occupancy at MYC/E2F target gene promoters would substantially strengthen the mechanistic model. Add a paragraph in the Discussion acknowledging that the mechanistic link to MYC/E2F remains correlative and that future ChIP-seq or CUT&RUN studies are needed to establish direct regulation. This is honest and does not diminish the study's contribution.**

We agree with the reviewer and have added sentences to the Discussion acknowledging that the mechanistic link to MYC/E2F remains correlative and that future ChIP-seq or CUT&RUN analysis of H3K4me3 or CXXC1 occupancy at MYC/E2F target gene promoters are needed to test direct regulation.

**7. Several figures are difficult to interpret as provided:
Figure 1A: The heatmap uses "z-scored differential mean dependency" but the color scale is not shown. Additionally, many cell lines are annotated with asterisks indicating significance (FDR ≤ 0.10), but the asterisks are not consistently visible or explained in the legend.**

We have addressed these corrections in Figure 1A.

**Figures 3 and 4: The violin-boxplot overlays are visually crowded. The horizontal dashed lines (GMM thresholds) are difficult to distinguish from other plot elements.**

We have increased the spacing between the violin plots in figures to reduce visual crowding and improve readability, and we have thickened the horizontal dashed lines marking the GMM-derived thresholds so that they are clearly distinguishable from the other plot elements.

**Supplementary Figures: Figure S1A-B are mentioned (Page 12, lines 25-28) but the panel labels in the PDF are incomplete (Page 40 shows a long string of "1"s instead of the actual heatmap). This appears to be a rendering error.
Provide high-resolution figures with clear color bars, legible fonts (minimum 8pt for axis labels), and consistent annotation. Ensure all supplementary figures render correctly. Consider using separate panels (e.g., 1Ai, 1Aii) for zoomed-in views of key complexes.**

We have reviewed all main and supplementary figures to ensure they render correctly in the final figures. Where possible, we increased font sizes to a minimum of 8 pt for axis labels and other figure text. We also carefully reviewed all figures and supplementary figures to ensure high resolution and image quality throughout the manuscript.

**8. Tighten redundant writing across Abstract, Summary, and Introduction. Fix typographical errors ("Plasmonic Prophylactic" → "Plasmocin Prophylactic"). Standardize hyphenation and formatting of statistical terms.**

We checked the original manuscript and confirmed that “Plasmocin Prophylactic” was spelled correctly. We are not sure how the reviewer encountered this error. Nevertheless, we carefully proofread the entire manuscript to identify and correct any typographical or formatting inconsistencies. We also revised the text to improve conciseness by removing redundancies and standardizing hyphenation and formatting of statistical terms.
