# Supplementary material for: Systematic multivariate analysis of chromatin complex dependencies reveals Set1C/COMPASS as a melanoma-enriched epigenetic vulnerability
Source: PLoS Comput Biol. 2026 Aug 27;22(8):e1014018. doi: 10.1371/journal.pcbi.1014018 (PMC13537691; doi:10.1371/journal.pcbi.1014018)
Supplement: Peer review history

# Peer review: Decision Letter 0

**DOI:** 10.1371/journal.pcbi.1014018.r001

**Authors:** Mark Alber, Jennifer Wilson

12 May 2026

Systematic multivariate analysis of chromatin complex dependencies reveals Set1C/COMPASS as a melanoma-enriched epigenetic vulnerability

PLOS Computational Biology

Dear Dr. Fallahi-Sichani,

Thank you for submitting your manuscript to PLOS Computational Biology. After careful consideration, we feel that it has merit but does not fully meet PLOS Computational Biology's publication criteria as it currently stands. Therefore, we invite you to submit a revised version of the manuscript that addresses the points raised during the review process.

Please submit your revised manuscript by Jul 12 2026 11:59PM. If you will need more time than this to complete your revisions, please reply to this message or contact the journal office at ploscompbiol@plos.org. When you're ready to submit your revision, log on to https://www.editorialmanager.com/pcompbiol/ and select the 'Submissions Needing Revision' folder to locate your manuscript file.

Please include the following items when submitting your revised manuscript:

* A letter that responds to each point raised by the editor and reviewer(s). You should upload this letter as a separate file labeled 'Response to Reviewers'. This file does not need to include responses to formatting updates and technical items listed in the 'Journal Requirements' section below.

* A marked-up copy of your manuscript that highlights changes made to the original version. You should upload this as a separate file labeled 'Revised Manuscript with Track Changes'.

* An unmarked version of your revised paper without tracked changes. You should upload this as a separate file labeled 'Manuscript'.

Kind regards,

Jennifer Wilson

Academic Editor

PLOS Computational Biology

Mark Alber

Section Editor

PLOS Computational Biology

**Additional Editor Comments:**

Reviewers agreed that the study was rigorous and interesting. Multiple reviewers noted that including more rationale for decisions and altering some discussion points is warranted.

**Journal Requirements:**

If the reviewer comments include a recommendation to cite specific previously published works, please review and evaluate these publications to determine whether they are relevant and should be cited. There is no requirement to cite these works unless the editor has indicated otherwise.

1) Please ensure that the CRediT author contributions listed for every co-author are completed accurately and in full.

At this stage, the following Authors/Authors require contributions: Luisa Quesada Camacho, and Mohammad Fallahi-Sichani. Please ensure that the full contributions of each author are acknowledged in the "Add/Edit/Remove Authors" section of our submission form.

The list of CRediT author contributions may be found here: https://journals.plos.org/ploscompbiol/s/authorship#loc-author-contributions

2) Please provide an Author Summary. This should appear in your manuscript between the Abstract (if applicable) and the Introduction, and should be 150-200 words long. The aim should be to make your findings accessible to a wide audience that includes both scientists and non-scientists. Sample summaries can be found on our website under Submission Guidelines:

https://journals.plos.org/ploscompbiol/s/submission-guidelines#loc-parts-of-a-submission

3) We do not publish any copyright or trademark symbols that usually accompany proprietary names, eg ©,  ®, or TM  (e.g. next to drug or reagent names). Therefore please remove all instances of trademark/copyright symbols throughout the text, including:

- TM on page: 18.

4) Please upload all main figures as separate Figure files in .tif or .eps format. For more information about how to convert and format your figure files please see our guidelines:

https://journals.plos.org/ploscompbiol/s/figures

5) When completing the data availability statement of the submission form, you indicated that you will make your data available on acceptance. We strongly recommend all authors decide on a data sharing plan before acceptance, as the process can be lengthy and hold up publication timelines. Please note that, though access restrictions are acceptable now, your entire data will need to be made freely accessible if your manuscript is accepted for publication. This policy applies to all data except where public deposition would breach compliance with the protocol approved by your research ethics board. If you are unable to adhere to our open data policy, please kindly revise your statement to explain your reasoning and we will seek the editor's input on an exemption. Please be assured that, once you have provided your new statement, the assessment of your exemption will not hold up the peer review process.

**Reviewers' comments:**

Reviewer's Responses to Questions

**Comments to the Authors:**

**Please note here if the review is uploaded as an attachment.**

Reviewer #1: In this study, Quesada Camacho and Fallahi-Sichani have systematically identified lineage-enriched epigenetic vulnerabilities across many cancer types by bioinformatic analysis of public datasets. In melanoma, they identified a novel dependence on the H3K4 methyltransferase complex Set1C/COMPASS. Using a panel of melanoma cell lines, the authors show that some but not all cell lines are dependent on CXXC1 with dependent cell lines showing reduced H3K4 trimethylation and cell cycle progression upon CXXC1 depletion by siRNA. They also show that CXXC1-dependent cell lines show alterations in E2F and MYC pathways upon CXXC1 depletion.

Overall, the study is well done and carefully controlled. The bioinformatic analysis is well done, and the experiments with CXXC1 knockdown are very thorough. In addition, the top-level finding that some but not all melanomas are dependent on the Set1C/COMPASS complex would be novel. However, there are several weaknesses that the authors should address to strengthen their findings:

- CXXC1 is a member of both the SETD1A and SETD1B COMPASS complexes. Thus, the data demonstrating that some cell lines are CXXC1-dependent does not distinguish between SETD1A and SETD1B. The authors should add experimental demonstration that the CXXC1-dependent cell lines are also sensitive to SETD1B knockdown.

- Despite the careful characterization of the effects of CXXC1 knockdown, there does not appear to be any way to identify CXXC1-dependent cell lines. In other words, what are the biomarkers of CXXC1-dependence? Or is it impossible to know until you knock down CXXC1 and measure the response?

- The epigenetic complex dependence analysis done by cancer lineage (Fig. 1) misses dependencies that are unique to cancer sub-types (e.g., BRCA can be divided into luminal A/B, HER2-positive, and basal). Have the authors explored whether there are sub-type-dependent epigenetic dependencies for those tumor types where there are sufficient number of cell lines?

- The variance explained by PC4 in Fig. 6B is only 2.7%. Can the authors comment on or justify why the effect of CXCC1 knockdown is so small relative to cell type effects?

- The authors use two somewhat arbitrary thresholds in the analysis of VIP scores in Fig. 2C (0.6 and 1.0). Is there a threshold-free analysis that could be done to reduce the reliance on these thresholds?

Minor Points

• Figure 6E has a typo in the title

Reviewer #2: Overview:

This paper leverages large public datasets to investigate epigenetic differences across cell lines representing many different types of cancer. This work is relevant because the ways in which epigenetic mechanisms regulate specific cancer types is not well studied. Analyses of the datasets identify a lineage-specific epigenetic vulnerability in melanoma that is independent of the melanoma differentiation state. Specifically, the authors find that the methyltransferase complex Set1C/COMPASS involving the CXXC1 subunit of SETD1B influences cell proliferation and transcriptional programs in CXXC1-dependent melanoma cell lines.

Overall, the paper provides relevant and interesting findings, the methods are rigorous and well justified, and the work is presented clearly. However, some aspects of the paper should be refined to improve the study.

Specific comments:

- My main concern is related to justifying the results and analyses presented. This is reflected in a number of ways:

1. Although the initial analysis included 42 cancer types, the paper almost immediately focused on melanoma. Greater justification is needed here, as many threads could be pulled on given the rich results shown in Fig. 1A. Why did melanoma specifically become the focus?

2. Similar to melanoma, in Fig. 1A, MBN and AML also have a strong dependency on Set1C/COMPASS. Should this be further investigated? Some discussion is needed here.

3. Is it meaningful that SETD1B and CXXC1 are only considered important contributors to the epigenetic gene-dependency when the VIP threshold is relaxed? More justification is needed.

4. When silencing CXXC1, the CXXC1-dependent cell line LOXIMVI does not show differences in Ki67 to the same extent as the other CXXC1-dependent lines, but does show differences in the percentage of Ki67-high cells compared to control (Fig. 3A and H). This cell line is also an outlier in Fig. 3J. The authors should explain this.

5. Related, the LOXIMVI cell line is not considered in the analyses presented in Fig. 4. What is the justification for this?

6. Only a portion of the transcriptional programs associated with Set1C/COMPASS dependency are highlighted in Fig. 5. What is the reasoning for this? Of particular interest are the programs related to EMT that are negatively associated (see comment below).

- As written, the single-cell analysis is disconnected from the other analyses. Can the authors better integrate these findings in the narrative? Is the greater cell-to-cell heterogeneity for different markers meaningful? How can the insights from single cells be leveraged?

- I find it quite interesting that the EMT genes are negatively associated with Set1C/COMPASS dependency (Fig. 5; Fig. 6F,G). Related, there is also a negative association with TGFbeta and TNF-A, which are related to EMT. The authors should discuss this finding and is relevance in melanoma.

- Minor comment: Perhaps I missed it, but what do the colors in the labels along the vertical and horizontal axes in Fig. 1A correspond to? These should be explained.

Reviewer #3: This manuscript presents a well-executed, integrative computational and experimental study identifying Set1C/COMPASS as a melanoma-enriched epigenetic vulnerability. The authors systematically analyze DepMap CRISPR dependency data across 42 cancer types, combined with curated chromatin complex annotations, to uncover lineage-specific dependencies at the complex level. They validate their findings through targeted siRNA experiments, multiplexed immunofluorescence imaging (4i), single-cell analysis, and RNA-seq. The work is timely, methodologically sound, and addresses an important gap in understanding context-dependent epigenetic dependencies in cancer. However, several issues regarding methodological transparency, statistical rigor, and presentation clarity should be addressed before publication.

1. The finding that Set1C/COMPASS supports MYC-driven transcriptional programs in cancer is not entirely new (see Izumi et al., Leukemia 2025, cited by authors). While the authors acknowledge this, the novelty would be strengthened by more explicitly delineating what is uniquely melanoma-specific about this dependency versus a more general phenomenon across SETD1B-dependent cancers. Additionally, the authors mention that "mutations in Set1C/COMPASS subunits are infrequent... and do not correlate with dependency scores" (Page 13-14, lines 8-10) — but do not explore whether expression levels of complex subunits or known regulators (e.g., Menin, WDR5-interacting proteins) correlate with dependency. This represents a missed opportunity to identify predictive biomarkers. Add an analysis correlating Set1C/COMPASS dependency with expression of complex subunits, known interactors, or chromatin states (e.g., baseline H3K4me3 levels) across melanoma cell lines. If no strong correlates exist, state this explicitly as an important unresolved question.

2. The authors classify cell lines as CXXC1-dependent based on normalized cell counts following siRNA treatment (Figure 3E). However, the threshold for "dependency" is not statistically defined. For LOXIMVI, the change appears modest (approximately 15-20% reduction), yet it is grouped with MALME3M and UACC62. What is the statistical justification? A formal interaction test (siRNA condition × cell line) or a predefined effect size threshold would improve rigor. Perform a two-way ANOVA with interaction term to formally test whether the effect of CXXC1 knockdown on cell count differs across cell lines. Report effect sizes (e.g., Cohen's d) and define dependency as a statistically significant reduction with effect size above a clinically/biologically meaningful threshold (e.g., >20% reduction, or FDR < 0.05 with |log2FC| > 0.3).

3. The authors use Gaussian mixture models (GMMs) to binarize single-cell expression into "High" vs. "Low" states (Page 29, lines 2-8). This is appropriate, but the thresholds are derived by pooling data across conditions (si-NTC and si-CXXC1) within each cell line. This approach assumes that the underlying distribution components are stable across conditions, which may not hold if knockdown shifts the entire population. If the knockdown causes a uniform leftward shift without changing variance, the GMM threshold based on pooled data may misclassify cells. Either (a) fit separate GMMs for each condition and report classification consistency, or (b) use a fixed threshold based on the control condition only (e.g., 95th percentile of control, or mean + 2SD) and justify this choice. At minimum, perform a sensitivity analysis showing that main conclusions (Figures 3F-J, 4A-D) are robust to the thresholding method.

4. The authors identify 26 epigenetic regulator genes with melanoma-enriched dependency using limma (FDR ≤ 0.05, mean difference > 0.1). This is appropriate. However, in Figure 1A, the complex-level analysis uses FDR ≤ 0.10 (Page 33, line 39) without justification for the more lenient threshold. The authors should either (a) apply the same FDR ≤ 0.05 threshold consistently, or (b) clearly justify the more lenient threshold for complex-level analysis (e.g., reduced statistical power due to averaging across subunits, or exploratory nature of complex-level discovery). Apply FDR ≤ 0.05 to complex-level analysis as a primary threshold. If key complexes (e.g., Set1C/COMPASS) no longer meet this threshold, present the FDR ≤ 0.10 results as "suggestive" or "nominal" and temper language accordingly in the Results and Discussion. Alternatively, provide power calculations demonstrating why FDR ≤ 0.10 is justified.

5. A key claim is that CXXC1 dependency reflects Set1C/COMPASS activity (Page 15, lines 15-16: "To test whether dependency on CXXC1 in melanoma cells arises from its role in supporting Set1C/COMPASS activity"). However, the authors only show correlative evidence that CXXC1 depletion reduces H3K4me3 and proliferation. They do not test whether the growth defect is rescued by (a) overexpression of wild-type CXXC1, or (b) co-expression of other complex subunits (e.g., SETD1B) or (c) a catalytically inactive mutant (to distinguish scaffolding vs. enzymatic functions). Without rescue, off-target effects of siRNA cannot be definitively excluded. This is not necessarily required for publication in a computational journal, but the authors should acknowledge this limitation explicitly in the Discussion and tone down causal language (e.g., change "shows that CXXC1-dependent melanoma cells require Set1C/COMPASS activity" to "suggests that..." or "is consistent with..."). Alternatively, include at least one orthogonal validation (e.g., CRISPR knockout with complementation, or a second independent siRNA pool).

6. The GSEA linking Set1C/COMPASS dependency to MYC/E2F targets (Figure 5) is compelling but correlative. The RNA-seq following CXXC1 knockdown (Figure 6) shows downregulation of these programs, but does not distinguish direct from indirect effects. ChIP-seq for H3K4me3 or CXXC1 occupancy at MYC/E2F target gene promoters would substantially strengthen the mechanistic model. Add a paragraph in the Discussion acknowledging that the mechanistic link to MYC/E2F remains correlative and that future ChIP-seq or CUT&RUN studies are needed to establish direct regulation. This is honest and does not diminish the study's contribution.

7.Several figures are difficult to interpret as provided:

Figure 1A: The heatmap uses "z-scored differential mean dependency" but the color scale is not shown. Additionally, many cell lines are annotated with asterisks indicating significance (FDR ≤ 0.10), but the asterisks are not consistently visible or explained in the legend. Figures 3 and 4: The violin-boxplot overlays are visually crowded. The horizontal dashed lines (GMM thresholds) are difficult to distinguish from other plot elements. Supplementary Figures: Figure S1A-B are mentioned (Page 12, lines 25-28) but the panel labels in the PDF are incomplete (Page 40 shows a long string of "1"s instead of the actual heatmap). This appears to be a rendering error.

Provide high-resolution figures with clear color bars, legible fonts (minimum 8pt for axis labels), and consistent annotation. Ensure all supplementary figures render correctly. Consider using separate panels (e.g., 1Ai, 1Aii) for zoomed-in views of key complexes.

8. Tighten redundant writing across Abstract, Summary, and Introduction. Fix typographical errors ("Plasmonic Prophylactic" → "Plasmocin Prophylactic"). Standardize hyphenation and formatting of statistical terms.

**********

**Have the authors made all data and (if applicable) computational code underlying the findings in their manuscript fully available?**

The PLOS Data policy requires authors to make all data and code underlying the findings described in their manuscript fully available without restriction, with rare exception (please refer to the Data Availability Statement in the manuscript PDF file). The data and code should be provided as part of the manuscript or its supporting information, or deposited to a public repository. For example, in addition to summary statistics, the data points behind means, medians and variance measures should be available. If there are restrictions on publicly sharing data or code —e.g. participant privacy or use of data from a third party—those must be specified.

Reviewer #1: Yes

Reviewer #2: Yes

Reviewer #3: Yes

**********

PLOS authors have the option to publish the peer review history of their article (what does this mean?). If published, this will include your full peer review and any attached files.

If you choose “no”, your identity will remain anonymous but your review may still be made public.

**Do you want your identity to be public for this peer review?**  For information about this choice, including consent withdrawal, please see our Privacy Policy.

Reviewer #1: No

Reviewer #2: No

Reviewer #3: No

**Figure resubmission:**

After uploading your figures to PLOS’s NAAS tool - https://ngplosjournals.pagemajik.ai/artanalysis, NAAS will process the files provided and display the results in the "Uploaded Files" section of the page as the processing is complete. If the uploaded figures meet our requirements (or NAAS is able to fix the files to meet our requirements), the figure will be marked as "fixed" above. If NAAS is unable to fix the files, a red "failed" label will appear above. When NAAS has confirmed that the figure files meet our requirements, please download the file via the download option, and include these NAAS processed figure files when submitting your revised manuscript.

**Reproducibility:**

To enhance the reproducibility of your results, we recommend that authors of applicable studies deposit laboratory protocols in protocols.io, where a protocol can be assigned its own identifier (DOI) such that it can be cited independently in the future. Additionally, PLOS ONE offers an option to publish peer-reviewed clinical study protocols. Read more information on sharing protocols at https://plos.org/protocols?utm_medium=editorial-email&utm_source=authorletters&utm_campaign=protocols

# Author comment: Author response to Decision Letter 1

**DOI:** 10.1371/journal.pcbi.1014018.r002

21 Jul 2026

Attachment Submitted filename: Response_to_Reviews_PCOMPBIOL-D-26-00353.docx

# Peer review: Decision Letter 1

**DOI:** 10.1371/journal.pcbi.1014018.r003

**Authors:** Christoph Kaleta, Jennifer Wilson

17 Aug 2026

Dear Prof. Fallahi-Sichani,

We are pleased to inform you that your manuscript 'Systematic multivariate analysis of chromatin complex dependencies reveals Set1C/COMPASS as a melanoma-enriched epigenetic vulnerability' has been provisionally accepted for publication in PLOS Computational Biology.

Before your manuscript can be formally accepted you will need to complete some formatting changes, which you will receive in a follow up email. A member of our team will be in touch with a set of requests.

Please note that your manuscript will not be scheduled for publication until you have made the required changes, so a swift response is appreciated.

IMPORTANT: The editorial review process is now complete. PLOS will only permit corrections to spelling, formatting or significant scientific errors from this point onwards. Requests for major changes, or any which affect the scientific understanding of your work, will cause delays to the publication date of your manuscript.

Should you, your institution's press office or the journal office choose to press release your paper, you will automatically be opted out of early publication. We ask that you notify us now if you or your institution is planning to press release the article. All press must be co-ordinated with PLOS.

Thank you again for supporting Open Access publishing; we are looking forward to publishing your work in PLOS Computational Biology.

Best regards,

Jennifer Wilson

Academic Editor

PLOS Computational Biology

Mark Alber

Section Editor

PLOS Computational Biology

***********************************************************

Congratulations on your revised manuscript. Overall, the reviewers felt that the new manuscript was much improved and addressed major concerns. One reviewer had an additional minor comment that can be address in the discussion. However, it did not warrant another round of revisions.

Reviewer's Responses to Questions

**Comments to the Authors:**

**Please note here if the review is uploaded as an attachment.**

Reviewer #1: Overall, the authors have done a satisfactory job of addressing our concerns.

However, while the authors provide additional computational analysis that strengthens the conclusion that CXXC1 dependency is associated more closely with SETD1B than SETD1A, they do not address the original concern that there is no experimental proof that a knockdown SETD1B matches a CXXC1 knockdown.

The authors should either do the SETD1B knockdown or at least mention this in the Discussion as a weakness of their results.

Reviewer #2: The authors have provided a thorough response to my comments. Based on these changes, I believe manuscript is stronger. I have no additional suggestions.

Reviewer #3: I recommend acceptance. The authors have fully addressed the reviewers' concerns with rigorous new statistical analyses and compelling experimental data, making the study suitable for publication.

**********

**Have the authors made all data and (if applicable) computational code underlying the findings in their manuscript fully available?**

The PLOS Data policy requires authors to make all data and code underlying the findings described in their manuscript fully available without restriction, with rare exception (please refer to the Data Availability Statement in the manuscript PDF file). The data and code should be provided as part of the manuscript or its supporting information, or deposited to a public repository. For example, in addition to summary statistics, the data points behind means, medians and variance measures should be available. If there are restrictions on publicly sharing data or code —e.g. participant privacy or use of data from a third party—those must be specified.

Reviewer #1: Yes

Reviewer #2: Yes

Reviewer #3: None

**********

PLOS authors have the option to publish the peer review history of their article (what does this mean?). If published, this will include your full peer review and any attached files.

If you choose “no”, your identity will remain anonymous but your review may still be made public.

**Do you want your identity to be public for this peer review?**  For information about this choice, including consent withdrawal, please see our Privacy Policy.

Reviewer #1: No

Reviewer #2: No

Reviewer #3: No

# Editor report: Acceptance letter

**DOI:** 10.1371/journal.pcbi.1014018.r004

**Authors:** Christoph Kaleta, Jennifer Wilson

PCOMPBIOL-D-26-00353R1

Systematic multivariate analysis of chromatin complex dependencies reveals Set1C/COMPASS as a melanoma-enriched epigenetic vulnerability

Dear Dr Fallahi-Sichani,

I am pleased to inform you that your manuscript has been formally accepted for publication in PLOS Computational Biology. Your manuscript is now with our production department and you will be notified of the publication date in due course.

The corresponding author will soon be receiving a typeset proof for review, to ensure errors have not been introduced during production. Please review the PDF proof of your manuscript carefully, as this is the last chance to correct any errors. Please note that major changes, or those which affect the scientific understanding of the work, will likely cause delays to the publication date of your manuscript.

Soon after your final files are uploaded, unless you have opted out, the early version of your manuscript will be published online. The date of the early version will be your article's publication date. The final article will be published to the same URL, and all versions of the paper will be accessible to readers.

For Research, Software, and Methods articles, you will receive an invoice from PLOS for your publication fee after your manuscript has reached the completed accept phase. If you receive an email requesting payment before acceptance or for any other service, this may be a phishing scheme. Learn how to identify phishing emails and protect your accounts at https://explore.plos.org/phishing.

Thank you again for supporting PLOS Computational Biology and open-access publishing. We are looking forward to publishing your work!

With kind regards,

Sharmila Kamatchi

PLOS Computational Biology | Carlyle House, Carlyle Road, Cambridge CB4 3DN | United Kingdom ploscompbiol@plos.org | Phone +44 (0) 1223-442824 | ploscompbiol.org | @PLOSCompBiol
